# *Echinacea angustifolia* and *Echinacea purpurea* Supplementation Combined with Vaginal Hyaluronic Acid to Boost the Remission of Cervical Low-Grade Squamous Intraepithelial Lesions (L-SILs): A Randomized Controlled Trial

**DOI:** 10.3390/medicina58050646

**Published:** 2022-05-09

**Authors:** Gaetano Riemma, Maria Teresa Schettino, Gaetano Maria Munno, Diego Domenico Fasulo, Lucia Sandullo, Emanuele Amabile, Marco La Verde, Marco Torella

**Affiliations:** Obstetrics and Gynecology Unit, Department of Woman, Child and General and Specialized Surgery, University of Campania “Luigi Vanvitelli”, 80138 Naples, Italy; gaetano.riemma@unicampania.it (G.R.); mariateresa.sche@gmail.com (M.T.S.); gmm9401@gmail.com (G.M.M.); diegodomenico1993@gmail.com (D.D.F.); luciasandullo@virgilio.it (L.S.); amabilema93@gmail.com (E.A.); marco.laverde88@gmail.com (M.L.V.)

**Keywords:** *Echinacea angustifolia*, *Echinacea purpurea*, HPV, CIN-1, L-SIL

## Abstract

*Background and Objectives*: *Echinacea angustifolia* and purpurea have known immunomodulatory effects which boost viral clearance, including HPV infection. However, evidence regarding the improvement due to Echinacea-based supplements of cervical HPV-related pathologies is still lacking. The aim of this study is to evaluate the efficacy of Echinacea supplementation on the remission of cervical low-grade squamous intraepithelial lesions (L-SIL). *Materials and Methods*: A single-blind 1:1:1 parallel randomized controlled trial was conducted at the Colposcopy Unit of a tertiary care referral center. Reproductive-aged women were allocated either to (a) an oral supplement based on Echinacea extracts plus vaginal hyaluronic acid-based soft gel capsules, (b) the Echinacea supplement alone, or (c) vaginal hyaluronic acid-based soft gel capsules alone for 3 months. The primary outcome was the regression of cervical intraepithelial neoplasia (CIN)-1 for each treatment arm at 3, 6 and 12 months after the diagnosis. Secondary outcomes included changes in the epithelialization, pap smear, colposcopic parameters, histological reports, and vaginal health indexes (VHI) in the study groups. *Results*: 153 women (52 for arm A, 50 for arm B and 51 for arm C) completed the follow-up and were included in the analysis. There were no significant differences in both primary and secondary outcomes for the three groups after 3 months. At the 6-month follow-up, the number of persistent CIN-1 diagnoses was significantly lower in arm A (15/51), rather than in arm B (23/48, *p* = 0.03) and C (27/49, *p* = 0.03). Similarly, the same effect was seen after 12 months for treatment A (5/51) relative to B (15/48, *p* = 0.03) and C (14/48, *p* = 0.03). Colposcopic, histological and vaginal parameters were all significantly improved at 6 and 12 months for arm A relative to B and C, while no beneficial effects were seen after 3 months. *Conclusions*: Echinacea extracts supplementation in women with L-SIL/CIN-1 significantly boosts HPV lesion clearance, reducing the overall amount of diagnosis, histological, colposcopic and vaginal parameters after 6 and 12 months. However, a limited sample size reduces the quality of evaluated evidence, emphasizing the need for additional studies to validate these findings.

## 1. Introduction

Human papillomavirus (HPV) is the first-ranked sexually transmitted infection among reproductive-aged women [1]. It plays a pivotal role in the pathogenesis of cervical carcinoma, since almost all cervical malignant lesions are linked to persistent HPV infections [2]. Squamous carcinoma is the most diagnosed cervical cancer histotype. The original precancerous lesion of squamous carcinoma is known as “squamous intraepithelial lesion” (SIL) or “cervical intraepithelial neoplasia” (CIN). Such a lesion is categorized into two groups depending on the grade of intraepithelial neoplasia: low-grade (L-SIL/CIN-1) and high-grade (H-SIL/ CIN2−3) [3].

While high-grade lesions require intervention after histologic confirmation (e.g., large loop excision of the transformation zone, cryotherapy, or cold knife conization), low-grade lesions may only require follow-up, due to a low risk of progression to high-grade lesions and cancer [4].

In such a scenario, it has been hypothesized that the use of immunomodulatory supplements might improve the number of regressed lesions [5]. 

*Echinacea angustifolia* (EA) and *Echinacea purpurea* (EP) roots and extracts have been proven to show anti-inflammatory and immunostimulatory activities in several in vitro and in vivo studies [6]. Such trials were carried out to confirm the evidence available from the use of EA and EP in traditional Chinese medicine [7]. Initial studies showed that not every Echinacea extract has significant antiviral activity; however, only the above-ground portions, as well as the roots of EP, had significant antiviral activity, with evidence against H1N1 influenza virus, herpes simplex virus, and coronaviruses [8]. However, the antiviral effects of EP appeared less effective against intracellular pathogens, while viral particles located in the extracellular fluids appeared to be vulnerable to EP activity [9]. For these reasons, EP could perform a significant role during the first instance with HPV, which are the first phases of the infection (i.e., cervical metaplasia) [10].

Moreover, it has been seen that the roots, leaves, stems, and flowers of EP and EA could inhibit the expression of several proinflammatory cytokines, including interleukin (IL)-6 and IL-8, which are an expression of an induced proinflammatory state and are recognizable as proinflammatory markers [11]. Since EP and EA can downregulate both IL-6 and IL-8, its action might be involved in the modulation of an inflammatory state [11]. For this reason, Echinacea extracts cannot be considered clearly “immunostimulant” or “immune system booster,” since there is still a lack of evidence; however, they show a significant immunomodulatory action, which could justify their use against viruses, including HPV [12].

To date, the application of vaginal hyaluronic acid represents one of the most commonly used approaches to improve the restoration and re-epithelization of the cervix and to help spontaneous viral clearance. It is used as a standard treatment in many countries for treating L-SILs. However, some women experience prolonged persistence, and even progression, of the cervical lesion with the application of hyaluronic acid alone [13]. For this reason, some authors have proposed a combined approach, adjuvating the local agent with an oral immunomodulatory supplement [14].

The aim of the present study was to evaluate the efficacy of an adjuvant compound with a formulation based on 200 mg of an EA and EP supplement, HPVADL18^®^ (equal to 4 mg polyphenols plus 0.6 mg of echinacoside), on the regression of a CIN-1/L-SIL in patients undergoing a non-surgical approach.

## 2. Materials and Methods

This study was designed as a single-blind randomized controlled trial carried out at the Colposcopy and Cervical-Vaginal Pathology outpatient clinic of a tertiary care referral center (Obstetrics and Gynecology Unit of the AOU L. Vanvitelli, Naples, Italy) related to the University of Campania “Luigi Vanvitelli” between December 2019 and December 2021.

The Institutional Review Board (IRB) of the University of Campania “Luigi Vanvitelli” approved the study with protocol no. 728-26/11/19. All patients signed a written informed consent describing the therapeutic approach and randomization process, common side effects, and adverse reactions as well as privacy and anonymity protection that was guaranteed all along with the data collection and analysis.

Every procedure was carried out in accordance with the ethical standards of the institutional and national research committee and according to the 1964 Helsinki Declaration and its later amendments or comparable ethical standards. 

### 2.1. Inclusion and Exclusion Criteria

Women aged between 18 and 40 years of any ethnicity, with positivity to HPV testing (Cobas 4800 HPV Test, Roche Molecular Systems, Pleasanton, CA, USA) and a cytologic or histological (punch biopsy) diagnosis of CIN-1/L-SIL performed at the time of enrollment, with no contraindications to the proposed treatments and with an absence in their recent medical history (<3 months) for any type of pharmacological or nonpharmacological aid that could indirectly or directly modify the outcomes of interests, were considered eligible for the study. 

Women with an H-SIL cytologic diagnosis or CIN 2–3 histologic diagnosis at punch biopsy as well as microinvasive or invasive cervical carcinoma patients with a previous anti-HPV vaccination, pregnant patients, or women under immunosuppressive treatment as well as patients with human immunodeficiency virus infection were considered ineligible for the trial.

### 2.2. Sample Size

The sample size amount was calculated considering that, as reported in the current literature [15], about 60% of L-SILs could spontaneously regress without any treatment over 12-24 months, and this value is also higher in the case of positive HPV tests only. Assuming a significant increase of these regressions of at least 15% with at least one adjuvant therapy approach, and considering an alpha-error of 5% with a power of 80%, the sample size was at least 41women for each arm. Considering a 10% rate of patient drop-out, a minimum amount of 49 patients for the arm was considered necessary. 

### 2.3. Treatment

All the enrolled women completed a full clinical examination and subsequent follow-up for 12 months; the gynecological and physical examination included a Pap smear with an HPV test, and an evaluation of vaginal health (presence of leucorrhea, vaginal pH > 4:5, the positivity of the Whiff test)

Colposcopy and vulvoscopy were performed after an application of 3% acetic acid. Visible acetowhite lesions have been classified in accordance with the criteria of the International Federation of Cervical Pathology and Colposcopy [16]. 

After the examination, patients were randomly allocated 1:1:1 by the center according to the permuted block method, and the allocation sequence list was generated by computing random numbers. Only patients remained blinded to the content of the oral supplement or vaginal capsules as well as to the treatment allocation throughout the trial.

The treatment arms were divided as follows:

- Arm (A) vaginal hyaluronic acid-based soft gel capsules (Tiagin softgel, IDI Farmaceutici, Italy) once a day for 10 days for a 3-month period, adjuvated by a supplement containing *Echinacea angustifolia* (100 mg), *Echinacea purpurea* (100 mg), polyphenols (4mg), Vitamin C (40 mg) zinc (5 mg), copper (0.5 mg) (Normoimmuno, ADL Farmaceutici SRL, Italy) administered twice a day for 3 months.
- Arm (B) vaginal hyaluronic acid-based soft gel capsules (Tiagin softgel, IDI Farmaceutici, Italy) once a day for 10 days for a 3-month period.
- Arm (C) *Echinacea angustifolia* (100 mg), *Echinacea purpurea* (100 mg), polyphenols (4mg), vitamin C (40 mg) zinc (5 mg), copper (0.5 mg) (Normoimmuno, ADL Farmaceutici SRL, Italy) twice a day for 3 months.

**Figure 1:**
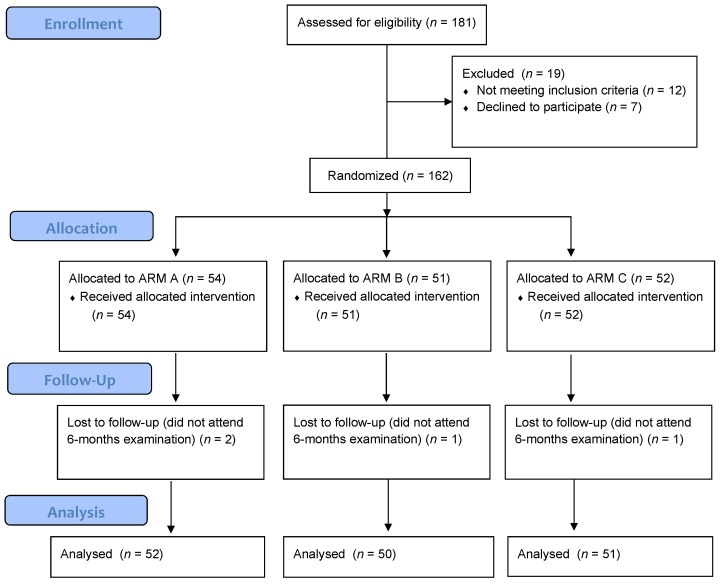
CONSORT Flow diagram for enrollment, allocation, follow-up, and analysis of women included in the trial.

The flow of patients through the study is depicted in Figure 1. 

Histological samples from targeted biopsies were obtained under colposcopic vision using Schumacher biopsy forceps and then analyzed according to a standardized procedure. Biopsy specimens were fixed in buffered formalin for 6 h. Subsequently, they were dehydrated using an automatized procedure, and they were embedded in paraffin after 24 h. Four μm-thick sections were obtained from paraffin-embedded tissue blocks. Routine hematoxylin-eosin staining was performed through an automatized procedure. 

### 2.4. Outcomes

The primary outcome of the study was the regression of CIN-1 for each treatment arm. While we included for enrollment both cytological and histological CIN-1 diagnoses, we considered the histological diagnosis of CIN-1 for the primary outcome at the 3 months follow-up, while the cytologic CIN-1 diagnosis was evaluated at the 6-months follow-up. Women with the persistence of CIN-1 after 6 months of follow-up were histologically re-evaluated at 12 months for lesion regression, persistence, or progression. Secondary outcomes were the evaluation of changes in the positivity and negativity of Pap smear classification (collected according to the Bethesda Classification System [17]), acetic acid and Lugol tests, histological reports, and vaginal health in the study groups.

### 2.5. Statistical Analysis

Statistical analysis was performed using Stata 14.1 (StataCorp LLC, College Station, TX, USA). Data were shown as mean with standard deviation (SD) or number (percentage). In addition, Chi-square or Fischer’s test for multiple comparisons was performed for categorical variables where appropriate. We performed a one-way analysis of variance (ANOVA) followed by a pairwise comparison using Tukey’s honestly significant difference (HSD) post hoc test for continuous variables. Statistical significance was defined as a *p*-value of (*p*) < 0.05.

## 3. Results

All 162 women were subjected to the assigned treatment. Two patients in arm A, one in arm B, and two patients in arm C did not attend the 6-month follow-up visit and were excluded from the analysis. Overall, 153 women were included in the analysis. A total of 52 patients underwent vaginal soft gel capsules with a nutritional supplement (arm A), 50 were subjected to vaginal capsules only (arm B), while for 51 patients, the nutritional supplement was administered alone (arm C).

There were no significant differences in demographic and baseline clinical characteristics between the study arms (Table 1).

### 3.1. 3 months Follow-Up

Data concerning the three treatment arms at the 3, 6, and 12 months follow-up are reported in Table 2 and Table 3.

There were no significant differences in the number of persistent histological CIN-1s upon cervical biopsies, as well as regressed and progressed (Table 2), and for each vaginal, cytologic, and colposcopic (Table 3) outcome of interest at the three months evaluation.

### 3.2. 6 months Follow-Up

Meanwhile, after 6 months, the number of persistent cytologic CIN-1 diagnoses was significantly lower in arm A compared to B (15/51 vs. 23/48, *p* = 0.03) and C (15/51 vs. 27/49, *p* = 0.03) (Table 2). In addition, the number of regressed lesions was significantly higher with the combined treatment (36/51) relative to vaginal hyaluronic acid (23/48, *p* = 0.03) or Echinacea (21/49; *p* = 0.03).

Similarly, women treated with EP and EA oral supplements plus vaginal capsules, rather than the EP supplementation or the capsule alone, showed improved vaginal, colposcopic, and cytologic indexes (Table 3).

### 3.3. 12 months Follow-Up

After 12 months of follow-up, the number of women without the persistence of a histological CIN-1 was still lower with the double therapy combination compared to the Echinacea extracts or vaginal hyaluronic acid alone (Group A (5/51) vs. B (15/48, *p* = 0.03) vs. C (14/48, *p* = 0.03)) (Table 2). On the other hand, the regression rate was significantly higher with the administration of EP and EA with vaginal hyaluronic acid (46/51) relative to the Echinacea extracts (32/38, *p* = 0.03) or vaginal hyaluronic acid (34/48, *p* = 0.03). At the same time, a significant improvement in vaginal, cytologic, and colposcopic parameters was observed in women of group A relative to B and C (Table 3).

No side or adverse effects were reported by the women of each group during the administration of the three treatment arms.

## 4. Discussion

These single-blind randomized controlled trials, which involved reproductive-aged women with a CIN-1 diagnosis, showed that adding a supplement based on EP and EA to a standard vaginal hyaluronic acid treatment reduces the number of remaining CIN-1 samples and shows better histological, cytologic, and colposcopic outcomes relative to vaginal hyaluronic acid or EP plus EA alone.

Several in vivo and in vitro studies have shown the immunomodulatory effects of Echinacea extracts. Park et al. [18] found that the oral intake of EP increased MHC II, CD4+ T cells, Th1 cytokines levels as well as NK cell activity. Moreover, the treatment increased B cell proliferation, leukocyte counts, and immunoglobulin levels, showing its capability of acting in immunomodulation systems [18].

In a recent prospective study, De Rosa et al. [19] showed that, in the case of HPV-related genital condylomatosis, the relapse incidence of visible warts was significantly lower with treatment with oral EP and EA and progressively decreases during the 12 months follow-up. The efficacy was for both men and women with visible condylomatosis. However, the efficacy was reduced in the case of extended condylomatosis, for which an increased relapse rate was reported. Such findings confirmed that EP and EA dry root extracts could be a valid adjuvant therapy to reduce the recurrence of genital condylomatosis [19].

However, it should be emphasized that, in this trial, the use of EP and EA alone did not prove significant advantages. Only combining the oral supplementation with the administration of the hyaluronic acid soft gel caps seemed to boost viral clearance and reduce the persistence of L-SIL/CIN-1 lesions during the 12-month follow-up.

The addition of zinc is important for regulating the immune system and stimulating the activity of leucocytes and NK cells. Concerning HPV-related lesions, both oral and topical zinc have been found significantly useful for treating cutaneous and genital condylomatosis. It has been shown that a lack of zinc is common in women with multiple or recurrent warts. It has been used alone as well as in combination with other treatments, including imiquimod, podophyllin, and cryotherapy [20].

Moreover, polyphenols have also shown interesting outcomes in avoiding cervical cancer progression. Resveratrol and pterostilbene showed promising anti-cancer activity and in vivo anti-tumor potential by downregulating the HPV oncogenic proteins E6 and E7 [21,22]. Similarly, polyphenolic constituents of tea (*Camellia sinensis*) demonstrated anti-cancer potential against cervical cancer, acting as an inhibitor of cell growth and apoptosis-inducer in HPV-16 positive human cervical cancer cells [23]. However, there is still a lack of in vivo studies and broader trials to validate these preliminary studies [21].

Other plant-based compounds showed interesting activity against HPV-related cervical lesions. The use of vaginally administered carboxy-methyl-beta-glucan showed a positive impact in the reduction of persistence and progression of L-SIL/CIN-1 lesions with at least 3 months of treatment [24]. Moreover, the use of Myrtus communis L-derived essential oils has shown promising restorative effects when used in a local treatment for HPV-positive women, thanks to its antimicrobial and antioxidant activity [25]. A herbal regimen based on curcumin, in which antioxidant activity is ascertained, has shown in vitro cytotoxicity against cervical cancer cells (throughout E6 and E7 inhibition) and it can be used as an adjuvant oral therapy to increase viral clearance and improve the regression of L-SILs [26]. Conversely, beta-carotene did not impact the regression of both L-SILs and H-SILs [27].

This study, involving the impact of EP and EA on L-SIL regression, has several strengths. Firstly, it is the first randomized trial on the research topic, avoiding the biases related to retrospective chart analyses. Secondly, the strict adhesion to the study protocol and the a priori decision of primary and secondary outcomes are additional points of strength.

However, this randomized trial shows several limitations. Primarily, we were not able to blind the operator to the treatment arms. Secondarily, the study protocol did not include a placebo arm to consider plausible nocebo effects. In addition, although the minimum sample size was reached, the number of patients for each treatment arm was still reduced, reducing the possibility of a generalization of the available results. In addition, it should be acknowledged that the increased frequency of cervical biopsies during follow-up could have affected the number of complete excisions of the lesion in women with persistent L-SILs.

## 5. Conclusions

This prospective randomized trial showed that adding an oral supplement of EP, EA, zinc, vitamin C, and polyphenols to vaginal hyaluronic acid soft gel capsules may boost the regression of a CIN-1/L-SIL and improve colposcopic, vaginal, and cytologic parameters compared to the use of the supplement or the capsules alone. However, due to the study limitations, further evidence is needed to validate the current findings.

## Figures and Tables

**Table 1:** Baseline characteristics of women included in treatment arms.

	Group A (52)	Group B (50)	Group C (51)	*p* Value	
Age, mean years (SD)	32.1 (4.3)	30.9 (3.9)	33.4 (2.8)	A vs. B A vs. C B vs. C	0.645 0.514 0.639
BMI, mean (SD)	25.2 (2.8)	24.9 (3.4)	23.8 (3.1)	A vs. B A vs. C B vs. C	0.883 0.812 0.795
Parity, mean (SD)	1.4 (0.9)	1.9 (1.2)	1.7 (1.5)	A vs. B A vs. C B vs. C	0.762 0.699 0.821
Smoking history, *n*.	34	29	26	A vs. B A vs. C B vs. C	0.579 0.612 0.726
Alcohol habits	12	9	15	A vs. B A vs. C B vs. C	0.335 0.251 0.209
Sexual partners (>4). *n*	21	19	18	A vs. B A vs. C B vs. C	0.771 0.832 0.769
CIN-1/L-SIL, *n*	52	50	51	A vs. B A vs. C B vs. C	0.849 0.914 0.901

SD: standard deviation.

**Table 2:** Regression, persistence, and progression of CIN-1/L-SILs during the 12-month follow-up.

	Group A	Group B	Group C	*p*-Value	
3 months histology					
CIN-1/L-SIL Regression	3/52 (5.8)	2/50 (4.0)	4/51 (7.9)	A vs. B A vs. C B vs. C	0.754 0.676 0.777
CIN-1/L-SIL Persistence	48/52 (92.3)	46/50 (92.0)	45/51 (88.2)	A vs. B A vs. C B vs. C	0.810 0.836 0.818
CIN-1/L-SIL Progression	1/52 (1.9)	2/50 (4.0)	2/51 (3.9)	A vs. B A vs. C B vs. C	0.602 0.799 0.812
6 months cytology					
CIN-1/L-SIL Regression	36/51 (70.6)	23/48 (47.9)	21/49 (42.8)	A vs. B A vs. C B vs. C	0.029 0.027 0.315
CIN-1/L-SIL Persistence	15/51 (39.4)	23/48 (47.9)	27/49 (55.1)	A vs. B A vs. C B vs. C	0.034 0.030 0.041
CIN-1/L-SIL Progression	0/51 (0)	2/48 (4.2)	1/49 (2.1)	A vs. B A vs. C B vs. C	0.555 0.629 0.212
12 months histology					
CIN-1/L-SIL Regression	46/51 (90.2)	32/48 (66.7)	34/48 (70.8)	A vs. B A vs. C B vs. C	0.026 0.025 0.581
CIN-1/L-SIL Persistence	5/51 (9.8)	15/48 (31.2)	14/48 (29.2)	A vs. B A vs. C B vs. C	0.027 0.029 0.614
CIN-1/L-SIL Progression	0/51 (0)	1/48 (2.1)	0/48 (0)	A vs. B A vs. C B vs. C	0.806 0.817 0.990

**Table 3:** Colposcopic, vaginal and cytologic outcomes at 3-, 6- and 12-months follow-up.

	3 months			6 months			12 months		
	A (*n* = 52)	B (*n* = 50)	C (*n* = 51)	A (*n* = 51)	B (*n* = 48)	C (*n* = 49)	A (*n* = 51)	B (*n* = 48)	C (*n* = 48)
Acetic acid Test, *n* (%)									
Positive	46 (88.4)	41 (82.0)	40 (78.4)	21 (43.1) *^,^°	30 (62.5)	29 (59.2)	10 (19.6) *^,^°	20 (41.7)	19 (39.6)
Negative	6 (11.6)	9 (18.0)	11 (22.6)	30 (56.9)	18 (37.5)	20 (40.8)	41 (80.4)	28 (58.3)	29 (60.4)
Lugol Test, *n* (%)									
Positive	40 (76.9)	41 (82.0)	42 (82.3)	25 (49.0) *^,^°	36 (75.0)	33 (67.3)	17 (33.3) *^,^°	29 (60.4)	27 (56.2)
Negative	12 (23.1)	9 (18.0)	9 (17.7)	26 (51.0)	12 (25.0)	16 (32.7)	34 (66.7)	18 (39.6)	21 (43.8)
Leucorrhea, *n* (%)									
Present	32 (61.5)	30 (60.0)	29 (56.8)	12 (23.5) *^,^°	15 (31.2)	17 (34.7)	10 (19.6) *^,^°	13 (27.1)	17 (35.4)
Absent	19 (38.5)	20 (40.0)	22 (43.1)	39 (76.5)	33 (68.8)	32 (65.3)	41 (80.4)	35 (72.9)	31 (64.6)
pH, *n* (%)									
<4.5	26 (50.0)	24 (48.0)	30 (58.8)	39 (76.5) *^,^°	30 (62.5)	31 (63.2)	40 (78.4) *^,^°	32 (66.7)	29 (60.4)
>4.5	26 (50.0)	26 (52.0)	21 (41.2)	12 (23.5)	18 (37.5)	18 (36.8)	11 (21.6)	16 (33.3)	19 (39.6)
Whiff test, *n* (%)									
Positive	24 (46.1)	25 (50.0)	25 (49.0)	12 (23.5) *^,^°	19 (39.5)	22 (44.9)	5 (9.8) *^,^°	15 (31.2)	20 (41.7)
Negative	28 (53.9)	25 (50.0)	26 (51.0)	39 (76.5)	29 (60.5)	27 (55.1)	46 (90.2)	33 (68.8)	28 (58.3)
Pap smear, *n* (%)									
Normal	9 (17.3)	8 (16.0)	10 (19.6)	35 (68.6) *^,^°	18 (37.5)	14 (28.6)	42 (82.3) *^,^°	30 (62.5)	23 (47.9)
Abnormal	43 (82.7)	42 (84.0)	41 (80.4)	26 (31.4)	30 (62.5)	35 (71.4)	7 (17.7)	18 (37.5)	25 (52.1)
Abnormal Pap smear subtype, *n* (%)									
ASCUS	17 (39.5)	15 (35.7)	15 (36.6)	13 (50.0)	12 (40.0)	13 (37.1)	4 (57.1)	10 (55.6)	15 (60.0)
L-SIL	23 (53.5)	22 (52.4)	22 (53.6)	13 (50.0)	16 (53.3)	19 (54.3)	3 (42.9)	8 (44.4)	7 (28.0)
ASC-H	2 (4.6)	3 (7.1)	3 (7.3)	0 (0)	0 (0)	3 (8.6)	0 (0)	0 (0)	1 (4.0)
AGC	1 (2.4)	2 (4.8)	0 (0)	0 (0)	2 (6.7)	0 (0)	0 (0)	0 (0)	2 (8.0)
H-SIL	0 (0)	0 (0)	1 (2.5)	0 (0)	0 (0)	0 (0)	0 (0)	0 (0)	0 (0)

* *p* < 0.05 vs. B; ° *p* < 0.05 vs. C.

## Data Availability

The data presented in this study are available on request from the corresponding author.
